# Dynamic contrast-enhanced magnetic resonance imaging of metacarpophalangeal joints reflects histological signs of synovitis in rheumatoid arthritis

**DOI:** 10.1186/s13075-014-0452-x

**Published:** 2014-10-01

**Authors:** Stefan Vordenbäumen, Christoph Schleich, Tim Lögters, Philipp Sewerin, Ellen Bleck, Thomas Pauly, Anja Müller-Lutz, Gerald Antoch, Matthias Schneider, Falk Miese, Benedikt Ostendorf

**Affiliations:** Department of Rheumatology, Heinrich-Heine-University, Moorenstraße 5, Düsseldorf, 40225 Germany; Department of Diagnostic and Interventional Radiology, University Dusseldorf, Medical Faculty, Moorenstraße 5, Düsseldorf, 40225 Germany; Department of Trauma and Hand Surgery, Heinrich-Heine-University, Moorenstraße 5, Düsseldorf, 40225 Germany; Department of Orthopaedics, River Rhein Center for Rheumatology at St. Elisabeth Hospital, Hauptstraße 74, Meerbusch-Lank, 40668 Germany

## Abstract

**Introduction:**

Synovial inflammation and joint destruction in rheumatoid arthritis (RA) may progress despite clinical remission. Dynamic contrast-enhanced magnetic resonance imaging (DCE-MRI) is increasingly used to detect synovial inflammation in RA. Although small joints such as metacarpophalangeal (MCP) joints are mainly affected by RA, MRI findings have never been directly compared to histological synovitis in MCP synovial tissue. The objective of the current study was therefore to analyse if DCE-MRI relates to histological signs of synovitis small RA joints.

**Methods:**

In 9 RA patients, DCE-MRI (3 Tesla, dynamic 2D T1 weighted turbo-flash sequence) of the hand was performed prior to arthroscopically-guided synovial biopsies from the second MCP of the imaged hand. Maximum enhancement (ME), rate of early enhancement, and maximum rate of enhancement were assessed in the MCP. Synovial biopsies were stained for determination of sublining CD68 and the Synovitis Score. Correlations between MRI and histological data were calculated according to Spearman.

**Results:**

ME of the MCP significantly correlated to sublining CD68 staining (r = 0.750, *P* = 0.02), the Synovitis Score (r = 0.743, *P* = 0.02), and the subscores for lining layer hypertrophy (r = 0.789, *P* = 0.01) and cellular density (r = 0.842; *P* = 0.004).

**Conclusions:**

Perfusion imaging of synovial tissue in RA finger joints employing DCE-MRI reflects histological synovial inflammation. According to our study, ME is the most closely associated parameter amongst the measures considered.

## Introduction

Rheumatoid arthritis (RA) is a debilitating disease characterized by chronic inflammation and proliferation of synovial tissue with subsequent destruction of cartilage and bone [1]. The target of modern therapeutic strategies consists of complete remission, which is commonly identified based on clinical grounds in conjunction with inflammatory markers such as C-reactive protein [2]. However, joint destruction may progress in patients thus considered to be in remission [3,4]. Hence, additional tools are needed to directly assess synovitis and cartilage destruction. Magnetic resonance imaging (MRI) is increasingly used for this purpose [5]. In particular, synovitis in MRI has been shown to relate to the histological degree of synovial inflammation in human RA [6,7] and arthritis models [8,9]. Generally, most correlative studies on MRI and synovial histology in RA were performed on large joints, especially knee joints [6,7], due to more easily accessible synovial tissue [10,11]. These findings are commonly extrapolated when MRI findings of small joints are assessed that are predominantly involved in RA, particularly in early disease states [12]. Data validating MRI findings by synovial histology of metacarpophalangeal (MCP) joints are so far scarce: gadolinium enhancement in synovial tissue correlated with macroscopic findings of hyperemia and vascularity, but synovial biopsies were not systematically assessed [13]. Data on dynamic MRI of MCP joints and corresponding histological findings are lacking. In the current study, we performed contrast-enhanced dynamic MRI of the hand with determination of perfusion parameters of synovial tissue of the MCP2 joint prior to arthroscopically guided synovial sampling of the same joint with histological analysis of synovial inflammation, with the aim to correlate dynamic MRI to histological synovitis.

## Methods

### Patients and synovial sampling

Nine patients with RA based on 2010 American College of Rheumatology/European League Against Rheumatism criteria with a 28-joint disease activity score (DAS28) >3.2, who required initiation of disease-modifying antirheumatic drug therapy (three patients, methotrexate) or a switch of medication (patients with methotrexate additionally received adalimumab (four patients), tocilizumab (one patient), or rituximab (one patient)) and gave their full informed written consent, were recruited into the study. After clinical examination (including DAS28, patient-reported ratings on 10-point scales for pain of the dominant MCP2 joint and global well-being, physician-rated 68-tender and swollen joint count), all patients received MRI of the more severely affected hand up to 1 week prior to arthroscopy-guided synovial sampling as described previously [10]. A total of six synovial biopsies were obtained from each patient under visual control from macroscopically inflamed areas and were snap frozen in Tissue-Tek (Sakura Finetek Germany, Staufen, Germany). The study was conducted according to the principles expressed in the Declaration of Helsinki and was approved by the ethics committee of the Medical Faculty of Heinrich-Heine-University (study number 3390).

### Histological assessment of synovitis

The work-up and scoring of tissue sections was carried out in a blinded fashion as described previously [10]. Briefly, 3 to 5 μm sections were prepared from snap-frozen synovial tissue, hematoxylin and eosion stained (Merck, Darmstadt, Germany) and evaluated prior to immunohistochemical staining of parallel sections of a suitable biopsy including a lining layer with a monoclonal mouse anti-CD68 antibody (Dako, Glostrup, Denmark). Hematoxylin and eosion -stained sections were used for determination of the synovitis score according to Krenn and colleagues [14], which is a semiquantitative four-point sum scale considering lining layer hypertrophy, inflammatory infiltrate, and density of resident cells. For scoring of sublining CD68 staining, images were photographed at 100× magnification (Axioskop 2 plus; Carl Zeiss, Jena, Germany; and Nikon DS Vi 1; Nikon, Düsseldorf, Germany) and stored in TIF format (resolution of 1,600 × 1,200). ImageJ software [15] was used to select the sublining layer, and the image was thresholded to highlight the stained areas but not the respective isotype controls. The stained area was calculated as a fraction of the selected region.

### Magnetic resonance imaging

MRI was performed on a 3 T MRI system (Magnetom Trio; Siemens Healthcare, Erlangen, Germany). Perfusion imaging (dynamic contrast-enhanced MRI) was acquired with a dynamic two-dimensional T1-weighted turbo-flash sequence. Twenty seconds after the beginning of the sequence, the contrast agent Magnevist® (Gd-DTPA, Bayer Healthcare, Leverkusen, Germany) was injected at a dosage of 0.4 ml/kg body weight. The acquisition parameters of the dynamic contrast-enhanced MRI sequence were: repetition time = 333 milliseconds, echo time = 1.46 milliseconds, acquisition time per scan acquisition time = 1.7 seconds, flip angle = 8°, field of view = 120 × 120 mm, 200 dynamical images and five acquired slices with a slice thickness of 4 mm.

Perfusion analysis in MCP joint synovial tissue was assessed using semiquantitative analysis parameters calculated with T-One weighted Perfusion imaging Parameter CAlculation Toolkit software (TOPPCAT, Daniel P. Barboriak, Duke University School of Medicine, Durham, North Carolina, USA). In definite region of interest TOPPCAT analyses, the mean signal intensity (*S*(*t*)) over time was employed to calculate the maximum level of synovial enhancement (ME), the maximum rate of enhancement (MV) per second, and the rate of early enhancement (REE) 17 seconds after onset of synovial enhancement using the formulas:

$$ \mathrm{ME} = \mathrm{maximum}\left(S(t)\right) - \mathrm{minimum}\left(S(t)\right) $$

$$ \mathrm{M}\mathrm{V} = \mathrm{maximum}\left(S\left(i+1\right) - S\left(i\ \hbox{--}\ 1\right)\right)\ /\ \left(t\left(i+1\right) - t\left(i\ \hbox{--}\ 1\right)\right) $$

$$ \mathrm{R}\mathrm{E}\mathrm{E} = \left(S2\ \hbox{--} Si\right)/\left(Si \times 17\ \mathrm{seconds}\right) \times 100\% $$

where *Si* is the signal intensity at time point *ti*, S1 is the signal intensity at onset of synovial enhancement, and S2 is the signal intensity 17 seconds after onset of synovial enhancement. In our study, REE was calculated 17 seconds after onset of synovial enhancement because we acquired a dynamic image every 1.7 seconds. Thus, we used the first 10 breakpoints for REE calculation. In all patients, the region of interest was defined as the anatomical area corresponding to the synovial membrane based on contrast-enhanced T1 images.

### Statistical analysis

Correlations between MRI parameters (ME, REE, MV) and parameters of histological synovitis (Synovitis Score, sublining CD68 staining) or clinical data were calculated according to Spearman. *P* <0.05 was considered significant. SPSS 22 (IBM, Armonk, NY, USA) was used for analyses.

## Results

Nine patients (seven female, two male, age 57.5 ± 14.3 years) were recruited, all of whom adhered to the study protocol. Mean DAS28 at inclusion was 5.4 (range 3.5 to 7.4). In the follow-up, there were no severe adverse events within 6 months such as any permanent tissue damage, damage to nerves or vessels, infections, thrombosis, or embolisms following arthroscopy of the MCP2 joint. The median histologic Synovitis Score was 6 (range 1 to 9), corresponding to a high-grade synovitis (score >4 [14]) in seven of nine patients.

Next, we compared dynamic MRI findings with clinical characteristics of the patients. Patient-rated pain of the MCP2 joint on a 10-point scale correlated very strongly with ME (*r* = 0.848, *P* <0.005), and to a somewhat lesser extent with REE (*r* = 0.681, *P* = 0.04) and MV (*r* = 0.695, *P* = 0.04). No correlations were noted between the MRI parameters of the MCP2 and the global patient assessment on a 10-point scale, total tender or swollen joint count. Furthermore, a nonsignificant correlation between the DAS28 and ME (*r* = 0.661, *P* = 0.053) was noted.

**Figure 1:**
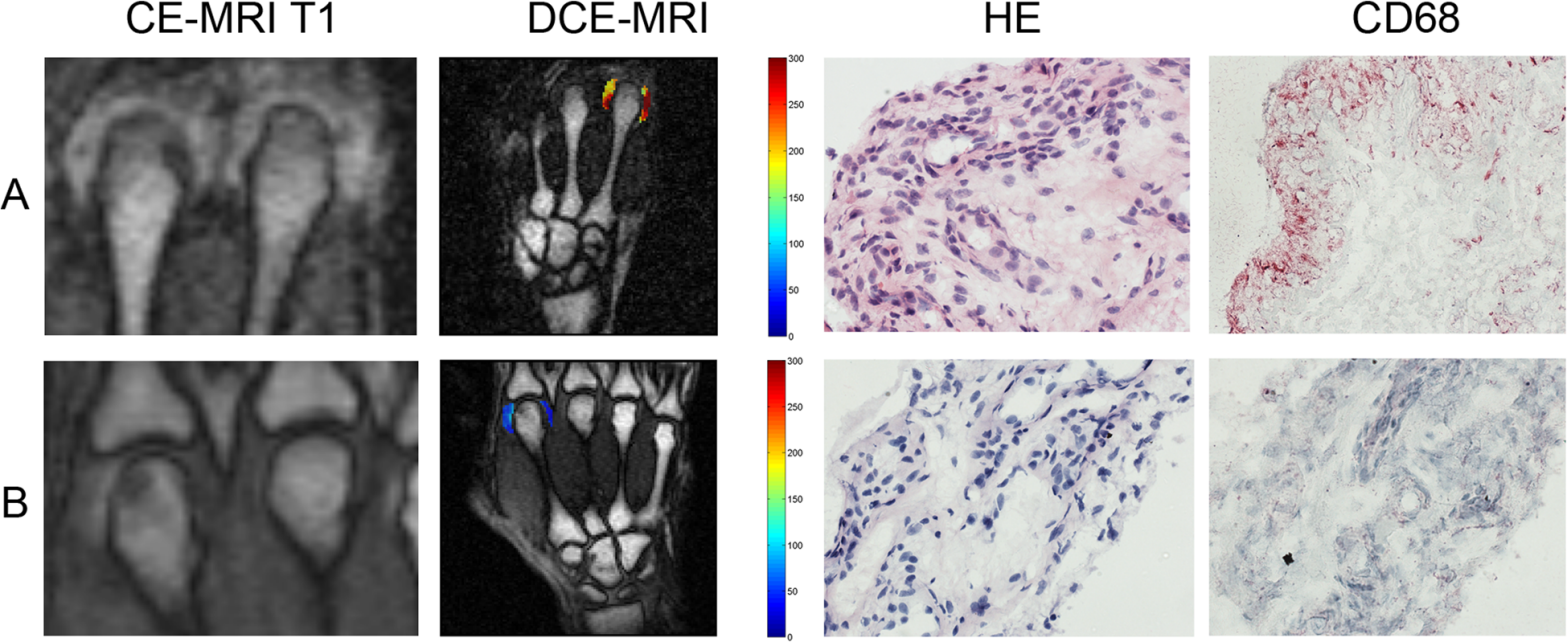
**Contrast-enhanced T1-weighted magnetic resonance imaging of metacarpophalangeal (MCP) joints 2 and 3 and maximum synovial enhancement of MCP 2.** Illustration of contrast-enhanced T1-weighted magnetic resonance imaging of metacarpophalangeal joint 2 and joint 3 (CE-MRI T1), fusion image of CE-MRI T1 and maximum synovial enhancement of MCP 2 measured by dynamic contrast-enhanced magnetic resonance imaging (DCE-MRI), and histological samples from the imaged joint stained with hematoxylin and eosion (HE) or immunohistochemical staining for macrophages (CD68). Patient with **(A)** a high degree of synovitis and **(B)** a low degree of synovitis. Red corresponds to a high maximum enhancement.

Finally, dynamic MRI parameters were compared with histological signs of synovitis by correlation analysis. As can be seen in Table 1 and is exemplified in Figure 1, strong correlations between ME and several histological measures of synovitis were present. No such correlations were found for either REE or MV.

**Table 1:** **Correlation between dynamic MRI and histological signs of synovitis in MCP2 joints according to Spearman**

	**ME**	**REE**	**MV**
Synovitis Score^a^	**0.743 (** ***P*** **= 0.02)**	0.228 (*P* = 0.56)	0.270 (*P* = 0.48)
Lining layer hypertrophy	**0.789 (** ***P*** **= 0.01)**	0.177 (*P* = 0.65)	0.346 (*P* = 0.36)
Inflammatory infiltrate	0.249 (*P* = 0.52)	0.053 (*P* = 0.89)	−0.107 (*P* = 0.78)
Cellular density	**0.842 (** ***P*** **= 0.004)**	0.408 (*P* = 0.28)	0.461 (*P* = 0.21)
Sublining CD68	**0.750 (** ***P*** **= 0.02)**	0.450 (*P* = 0.22)	0.567 (*P* = 0.11)

Significant results in bold. MCP, metacarpophalangeal; ME, maximum synovial enhancement; MRI, magnetic resonance imaging; MV maximum enhancement velocity; REE, rate of early enhancement. ^a^Synovitis Score according to Krenn and colleagues [14].

## Discussion

Recent developments in MRI technology are increasingly used to visualize all anatomical components of joints in RA down to a molecular level and permit functional imaging; for example, the assessment of synovial perfusion [16,17]. Findings such as bone marrow edema have prognostic value for future erosive disease [18]. Many of these findings have been validated on histological specimens, such as bone marrow edema and erosions [19,20], or synovial contrast enhancement and synovitis [6,7,9]. These studies have been performed in animal models or knee joints. However, RA has a predilection for small joints such as the MCP joints. Owing to a lack of other data, the validity of MRI findings of synovitis was hitherto extrapolated from large to small joints based on the abovementioned pioneering works. In the present study, we demonstrate that synovitis of MCP joints measured by ME on dynamic MRI strongly correlates to histological inflammation within the same joint. Besides conventional histological criteria, this finding extends to sublining CD68 staining, which is considered one of the best histological markers for disease activity in RA by many experts [21]. These data underscore the validity of dynamic contrast-enhanced MRI for the assessment of the degree of synovitis in small joints.

There are some limitations to this study. The small sample size is due to the invasiveness of the arthroscopic procedure and the resultant effort to keep the number of patients as small as possible with respect to the aim of the study. In spite of this, significant and consistent results were obtained. Moreover, additional synovial parameters could have been assessed such as markers of diverse cell populations and adhesion molecules. However, biopsies of MCP joints did not yield material in sufficient quality for multiple analyses in all cases. Of note, we applied a comparatively high dose of the contrast agent Magnevist® according to a standardized RA study protocol in our facility, which theoretically permits additional analyses such as delayed gadolinium-enhanced MRI [17].

## Conclusion

ME measured by dynamic MRI reflects histologic synovitis and may replace invasive sampling of synovial tissue in larger studies for the assessment of the degree of synovitis of small joints such as MCP. Our findings strongly support the use of dynamic MRI to assess synovitis in small joints of RA patients in a clinical setting.

## Abbreviations

DAS28: 28-joint disease activity score
MCP: metacarpophalangeal
ME: maximum synovial enhancement
MRI: magnetic resonance imaging
MV: maximum rate of enhancement
RA: rheumatoid arthritis
REE: rate of early enhancement
TOPPCAT: T-One weighted perfusion imaging parameter CAlculation toolkit
